# ClinicalTrials.gov as a Source of Information About Expanded Access Programs: Cohort Study

**DOI:** 10.2196/26890

**Published:** 2021-10-28

**Authors:** Jan Borysowski, Andrzej Górski

**Affiliations:** 1 Department of Clinical Immunology Medical University of Warsaw Warsaw Poland; 2 Centre for Studies on Research Integrity Institute of Law Studies Polish Academy of Sciences Warsaw Poland; 3 Laboratory of Bacteriophages Ludwik Hirszfeld Institute of Immunology and Experimental Therapy Polish Academy of Sciences Wrocław Poland

**Keywords:** ClinicalTrials.gov, expanded access, expanded access program, compassionate use, unapproved drug, investigational drug

## Abstract

**Background:**

ClinicalTrials.gov (CT.gov) is the most comprehensive internet-based register of different types of clinical studies. Expanded access is the use of unapproved drugs, biologics, or medical devices outside of clinical trials. One of the key problems in expanded access is the availability to both health care providers and patients of information about unapproved treatments.

**Objective:**

We aimed to evaluate CT.gov as a potential source of information about expanded access programs.

**Methods:**

We assessed the completeness of information in the records of 228 expanded access programs registered with CT.gov from February 2017 through May 2020. Moreover, we examined what percentage of published expanded access studies has been registered with CT.gov. Logistic regression (univariate and multivariate) and mediation analyses were used to identify the predictors of the absence of some information and a study’s nonregistration.

**Results:**

We found that some important data were missing from the records of many programs. Information that was missing most often included a detailed study description, facility information, central contact person, and eligibility criteria (55.3%, 54.0%, 41.7%, and 17.5% of the programs, respectively). Multivariate analysis showed that information about central contact person was more likely to be missing from records of studies registered in 2017 (adjusted OR 21.93; 95% CI 4.42-172.29; *P*<.001). This finding was confirmed by mediation analysis (*P*=.02). Furthermore, 14% of the programs were registered retrospectively. We also showed that only 33 of 77 (42.9%) expanded access studies performed in the United States and published from 2014 through 2019 were registered with CT.gov. However, multivariate logistic regression analysis showed no significant association between any of the variables related to the studies and the odds of study nonregistration (*P*>.01).

**Conclusions:**

Currently, CT.gov is a quite fragmentary source of data on expanded access programs. This problem is important because CT.gov is the only publicly available primary source of information about specific programs. We suggest the actions that should be taken by different stakeholders to fully exploit this register as a source of information about expanded access.

## Introduction

Expanded access, also termed compassionate use, is the use of unapproved drugs, biologics, or medical devices outside of clinical trials [1,2]. Regulations to permit expanded access to unapproved treatments have been introduced in many countries worldwide including the United States, many Member States of the European Union (EU), Canada, Australia, Japan, and Brazil [3-5]. In short, expanded access is a regulatory pathway that enables doctors to use unapproved treatments for patients with serious or life-threatening diseases who have run out of approved treatments and are not eligible for enrollment in a clinical trial [6].

ClinicalTrials.gov (CT.gov) is a comprehensive web-based repository of information about clinical studies performed both in the United States and other countries [7]. Originally, it was established to provide potential study participants with information about interventional clinical trials [8]. However, with time, other types of studies started to be registered with CT.gov, especially observational studies and expanded access programs. As of May 29, 2021, 758 expanded access programs have been registered with CT.gov [7]. According to the terminology adopted by CT.gov, those programs are termed expanded access studies; the latter term will be used throughout the article.

Importantly, not all types of studies have to be registered with CT.gov. Current regulations oblige the responsible parties to register, in particular, so-called applicable clinical trials, that is, trials meeting certain criteria. In addition, if the drug being investigated in an applicable clinical trial is available through expanded access and the responsible party in the trial is also the drug’s manufacturer, then the corresponding expanded access study has to be registered by statute as well [9]. However, there are no regulations to mandate the registration of observational studies.

Since expanded access involves the use of unapproved treatments, one of the key problems that can be encountered by both health care providers and patients is the availability of information about those treatments [10,11]. Access to data about unapproved treatments will certainly be more limited compared with standard approved therapies that are commonly known and used by many doctors. The objective of this study was to evaluate CT.gov as a source of information about expanded access studies. We focused on 3 main problems: (1) completeness of information required when registering an expanded access study with CT.gov, (2) mode of registration (prospective vs retrospective), (3) the percentage of expanded access studies that has been registered with CT.gov. These 3 problems are key in the evaluation of CT.gov because, to be helpful for patients and health care providers, CT.gov has to present complete data that are posted prospectively.

## Methods

### Selection of Expanded Access Studies Registered With ClinicalTrials.gov

Eligible studies were searched for in CT.gov [7] on June 8, 2020. Using the “Advanced Search” function, we selected expanded access studies involving the use of a drug, biologic, or medical device, registered with CT.gov from February 2017 through May 2020. We did not include studies registered by February 2017 because, in January 2017, some changes were introduced to the range of data that are required when registering expanded access studies with CT.gov. In particular, expanded access type and facility information started to be required in accordance with the Food and Drug Administration (FDA) Amendments Act (FDAAA) 801 final rule (42 CFR Part 11) [12].

### Extraction of Data From Records of ClinicalTrials.gov-Registered Expanded Access Studies

The record of each expanded access study registered with CT.gov includes a range of data classified either as required, conditionally required, or optional [12]. The range of data that we evaluated included unique protocol identification number; brief title; expanded access type; record verification date; expanded access status; responsible party, by official title; name of the sponsor; brief summary; detailed description; condition or focus of the study; intervention type; intervention name; eligibility including patient sex, age limits, and eligibility criteria; central contact person; facility information; citations to publications related to the expanded access; and links to web sites directly relevant to the expanded access.

Moreover, from the “History of Changes” field, we extracted the first recruitment status (“Available,” “No longer available,” “Temporarily not available,” or “Approved for marketing”).

### Publications on Expanded Access

We searched for publications on expanded access in Medline through Pubmed using the following search string: “Compassionate Use Trials” [MeSH] OR “expanded access” OR “compassionate use” OR “early access” OR “managed access” OR “named patient” OR “humanitarian device exemption.” The following inclusion criteria were used: (1) publication year 2014-2019; (2) a study involving the use of a drug, biologic, or medical device, with an explicit statement in the published article that the treatment was performed in an expanded accesss program or on a compassionate use basis; (3) at least 1 center located in the United States. We included expanded access studies regardless of the number of participants and study design (case studies, case series, and cohort studies; prospective and retrospective). The search for eligible publications was performed in May 2020.

For each of the included publications, we searched for a corresponding entry in CT.gov. This search was performed in 2 stages. First, we searched for an identifier typical of CT.gov using an automated search function (Ctrl-F). Since each CT.gov identifier starts with the prefix “NCT,” this prefix was used as the search term. In the second stage, for each publication that did not contain a CT.gov identifier, we searched CT.gov using keywords from the publication, especially intervention name and disease. This search was limited to expanded access studies performed in the United States (field “Country”). When assessing whether an article matched a study registered with CT.gov, we considered the type of study (expanded access), the intervention that was used, and center location.

### Statistical Analysis

Summary statistics were used to show absolute numbers and frequencies of main study characteristics. Different statistical tests were employed to evaluate whether the variables related to the expanded access studies are interrelated. These included a chi-square test (a discrete variable vs a discrete variable), Mann-Whitney test (a continuous variable vs a binary variable), analysis of variance (a continuous variable vs a discrete variable with more than 2 levels). For these tests, *P*<.05 was considered significant. Multicollinearity of the variables was assessed by determining the generalized variance inflation factor (GVIF). We considered that a GVIF value >5 may be indicative of multicollinearity.

Logistic regression (univariate and multivariate) analyses were performed to check whether posting of some data and registration of published expanded access studies depended on different variables. All variables with *P*<.05 in univariate analysis were entered into the multivariate model. For each regression analysis, the level of statistical significance was set using Bonferroni correction. The results of the logistic regression analyses were verified by mediation analysis. All computations were performed in R v. 3.6.1.

## Results

### Selection and Characteristics of Expanded Access Studies

**Figure 1:**
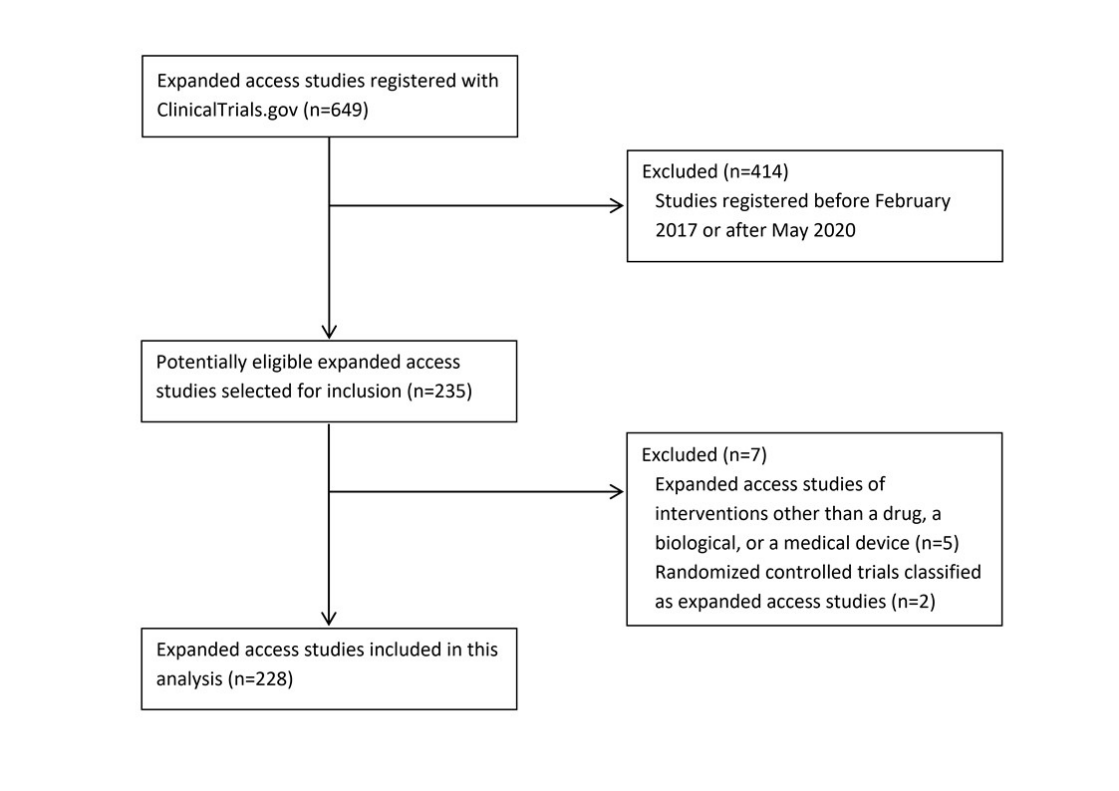
Flow diagram showing the selection of ClinicalTrials.gov-registered expanded access studies.

Eligible expanded access studies were identified in CT.gov [7]. The flow diagram showing the selection process is presented in Figure 1. We selected 228 studies registered with CT.gov from February 2017 through May 2020. The detailed characteristics of those studies are presented in Table 1. Overall, 195 different interventions were used in those studies. Most studies concerned oncology (96; 42.1%), neurology (29; 12.7%), or infectious diseases (28; 12.3%).

**Table 1:** Characteristics of the 228 ClinicalTrials.gov-registered, expanded access studies.

Characteristics		Number of studies, n (%)
**Medical specialty**		
	Oncology	96 (42.1)
	Neurology	29 (12.7)
	Infectious diseases	28 (12.3)
	Hematology	16 (7.0)
	Metabolic diseases	15 (6.6)
	Gastroenterology	8 (3.5)
	Cardiovascular diseases	7 (3.1)
	Pulmonology	5 (2.2)
	Other	17 (7.5)
	Unknown	7 (3.1)
**Intervention type**		
	Drug	162 (71.1)
	Biologic	61 (26.8)
	Medical device	5 (2.2)
**Sponsor**		
	Industry	182 (79.8)
	Nonindustry	32 (14.0)
	Mixed	14 (6.1)
**Center location**		
	United States	75 (32.9)
	International	16 (7.0)
	Other	14 (6.1)
	Unknown	123 (54.0)
**Expanded access type**		
	IP^a^	103 (45.2)
	IS^b^	44 (19.3)
	Tr. IND/Pr.^c^	46 (20.2)
	Mixed^d^	24 (10.5)
	Unknown	11 (4.8)
**Multicenter studies**		
	Yes	50 (21.9)
	No	55 (24.1)
	Unknown	123 (54.0)
**Multinational studies**		
	Yes	16 (7.0)
	No	91 (39.9)
	Unknown	121 (53.1)

^a^IP: individual patients.

^b^IS: intermediate-size population.

^c^Tr. IND/Pr.: treatment investigational new drug (IND)/protocol.

^d^Any combination of individual patients, intermediate-size population, and treatment IND/protocol.

### Assessment of Data Contained in Records of Expanded Access Studies

We evaluated the completeness of information that is required or optional when registering an expanded access study with CT.gov. Remarkably, detailed description, facility information, central contact person, and eligibility criteria were missing from the records of 126 (126/228, 55.3%), 123 (123/228, 54.0%), 95 (95/228, 41.7%), and 40 (40/228, 17.5%) studies, respectively (Table 2). Except for detailed description, all those data are required by CT.gov.

**Table 2:** ClinicalTrials.gov-registered expanded access studies (n=228) with different missing data.

Data missing from the record	Number of studies, n (%)
Detailed description^a^	126 (55.3)
Facility information^b^	123 (54.0)
Central contact person^b^	95 (41.7)
Eligibility criteria^c^	40 (17.5)
Sex of patients^c^	31 (13.6)
Expanded access type^b^	11 (4.8)
Conditions or focus of study^c^	7 (3.1)
Expanded access status^b^	0 (0)
Responsible party^b^	0 (0)
Name of the sponsor^b^	0 (0)
Brief summary^b^	0 (0)
Intervention type^b^	0 (0)
Intervention name^b^	0 (0)
Patient age limits^c^	0 (0)
Unique protocol identification number^b^	0 (0)
Brief title^b^	0 (0)
Record verification date^b^	0 (0)
Responsible party^b^	0 (0)

^a^Optional data.

^b^Required data.

^c^Data conditionally required.

In this analysis, as a comparator for expanded access studies, we used a random sample of 220 clinical trials registered with CT.gov in the same period of time. We found that detailed description, facility information, and central contact person were missing from the records of 88 (88/220, 40.0%), 6 (6/220, 2.7%), and 82 (82/220, 37.3%) trials, respectively. However, eligibility criteria, as well as all other types of required data were contained in each clinical trial record that we examined.

Using logistic regression analysis, we also determined the predictors of the absence of some data in the records of expanded access studies. This analysis was performed for detailed description, central contact person, and facility location (ie, the data that were missing from the records most often). The following variables were entered into the analysis: registration year, funding source, US involvement, whether the study was multicenter and multinational, and type of expanded access. Registration year is the year in which a study was registered with CT.gov (we examined studies registered between 2017 and 2020). Funding sources were divided into industry and nonindustry based on information contained in the “Sponsor” field of an expanded access study record (the category “Industry” included all for-profit organizations, especially pharmaceutical companies, while nonindustry sources included all non-for-profit organizations such as universities, academic reasearch centers, and federal agencies). The variable “US involvement” refers to the presence of at least 1 center located in the United States. A multicenter study was defined as a study performed at 2 or more centers. Studies performed in at least 2 countries were considered multinational. The type of expanded access was divided into 3 categories: (1) individual patients, (2) intermediate-size population, (3) treatment investigational new drug (IND)/protocol (ie, use of an unapproved treatment in a large population of patients). This division is in line with the FDA regulations [1] and was also adopted by CT.gov [12].

In this analysis, we adjusted for multiple comparisons using Bonferroni correction. The adjusted level of statistical significance was set at *P*=.002. In addition, prior to performing logistic regression analysis, we evaluated the multicollinearity of the variables. However, the GVIF value for each variable was between 1.0 and 1.7, indicative of a lack of multicollinearity (detailed data not shown).

Detailed results of the logistic regression analysis are shown in Table 3, Table 4, and Table 5. Univariate analysis showed that facility location was less likely to be missing from records of treatment IND/protocol studies related to individual patient expanded access studies (odds ratio [OR] 0.76; 95% CI 0.64-0.9; *P*=.001). Information about the central contact person was more likely to be missing in studies registered in 2017 (OR 1.53; 95% CI 1.26-1.86; *P*<.001) compared with those registered in 2020. Multivariate analysis showed that information about central contact person was more likely to be missing from records of studies registered in 2017 (adjusted OR [aOR] 21.93; 95% CI 4.42-172.29; *P*<.001).

**Table 3:** Predictors of the absence of study detailed description in the records of expanded access studies registered with ClinicalTrials.gov.

Variables		Univariate analysis		Multivariate analysis	
		Odds ratio (95% CI)^a^	*P* value	Adjusted odds ratio (95% CI)^a^	*P* value
**First posted date**					
	2020	referent	N/A^b^	referent	N/A
	2019	1.02 (0.84-1.23)	.84	N/A	N/A
	2018	1.12 (0.93-1.36)	.22	N/A	N/A
	2017	1.0 (0.81-1.23)	.98	N/A	N/A
**Sponsor**					
	Industry	referent	N/A	referent	N/A
	Nonindustry	0.76 (0.63-0.91)	.003	0.46 (0.19-1.08)	.07
**Multicenter study**					
	No	referent	N/A	referent	N/A
	Yes	1.08 (0.63-0.91)	.41	N/A	N/A
**Multinational study**					
	No	referent	N/A	referent	N/A
	Yes	0.93 (0.89-1.31)	.57	N/A	N/A
**US involvement**					
	No	referent	N/A	referent	N/A
	Yes	0.95 (0.71-1.21)	.66	N/A	N/A
**Type of expanded access**					
	IP^c^	referent	N/A	referent	N/A
	IS^d^	0.92 (0.75-1.2)	.33	0.75 (0.36-1.55)	.42
	Tr. IND/Pr.^e^	0.82 (0.77-1.09)	.02	0.47 (0.23-0.96)	.03

^a^For each variable (first posted date; sponsor; multicenter study; multinational study; US involvement; type of expanded access), odds ratios and 95% CI are shown related to the referent value.

^b^N/A: not applicable.

^c^IP: individual patients.

^d^IS: intermediate-size population.

^e^Tr. IND/Pr.: Treatment IND/protocol.

**Table 4:** Predictors of the absence of information about the central contact person in the records of expanded access studies registered with ClinicalTrials.gov.

Variables		Univariate analysis		Multivariate analysis	
		Odds ratio (95% CI)^a^	*P* value	Adjusted odds ratio (95% CI)^a^	*P* value
**First posted date**					
	2020	referent	N/A^b^	referent	N/A
	2019	1.17 (0.97-1.4)	.10	3.62 (0.8-26.08)	.13
	2018	1.26 (1.05-1.51)	.01	11.9 (2.5-89.64)	.004
	2017	1.53 (1.26-1.86)	<.001	21.93 (4.42-172.29)	<.001
**Sponsor**					
	Industry	referent	N/A	referent	N/A
	Nonindustry	0.89 (0.74-1.07)	.19	N/A	N/A
**Multicenter study**					
	No	referent	N/A	referent	N/A
	Yes	0.79 (0.65-0.95)	.01	0.33 (0.13-0.79)	.01
**Multinational study**					
	No	referent	N/A	referent	N/A
	Yes	0.83 (0.64-1.09)	.18	N/A	N/A
**US involvement**					
	No	referent	N/A	referent	N/A
	Yes	1.04 (0.83-1.31)	.73	N/A	N/A
**Type of expanded access**					
	IP^c^	referent	N/A	referent	N/A
	IS^d^	0.84 (0.71-1.0)	.05	N/A	N/A
	Tr. IND/Pr.^e^	0.95 (0.8-1.13)	.54	N/A	N/A

^a^For each variable (first posted date; sponsor; multicenter study; multinational study; US involvement; type of expanded access), odds ratios and 95% CIs are shown related to the referent value.

^b^N/A: not applicable.

^c^IP: individual patients.

^d^IS: intermediate-size population.

^e^Tr. IND/Pr.: Treatment IND/protocol.

**Table 5:** Predictors of the absence of information about the facility location in the records of expanded access studies registered with ClinicalTrials.gov.

Variables		Univariate analysis		Multivariate analysis	
		Odds ratio (95% CI)^a^	*P* value	Adjusted odds ratio (95% CI)^a^	*P* value
**First posted date**					
	2020	referent	N/A^b^	referent	N/A
	2019	0.83 (0.68-1.0)	.05	1.0 (0.9-1.1)	.94
	2018	1.0 (0.83-1.21)	.99	1.09 (0.98-1.22)	.10
	2017	0.85 (0.7-1.04)	.12	1.0 (0.9-1.11)	.98
**Sponsor**					
	Industry	referent	N/A	referent	N/A
	Nonindustry	0.89 (0.74-1.07)	.21	0.98 (0.9-1.07)	.65
**Multinational study**					
	No	referent	N/A	referent	N/A
	Yes	0.98 (0.91-1.05)	.55	N/A	N/A
**Type of expanded access**					
	IP^c^	referent	N/A	referent	N/A
	IS^d^	0.84 (0.71-1.0)	.05	0.99 (0.91-1.08)	.85
	Tr. IND/Pr.^e^	0.76 (0.64-0.9)	.001	1.04 (0.96-1.13)	.30

^a^For each variable (first posted date; sponsor; multicenter study; multinational study; US involvement; type of expanded access), odds ratios and 95% CIs are shown related to the referent value.

^b^N/A: not applicable.

^c^IP: individual patients.

^d^IS: intermediate-size population.

^e^Tr. IND/Pr.: Treatment IND/protocol.

In order to verify the results of the logistic regression analysis, we checked whether the variable “Registration date” was related with the other variables. Indeed, we found that this variable was related to the variable “Type of expanded access” (*P*=.002). By contrast, the relationships between “Registration date” and all the remaining variables were not statistically significant (*P*>.05). Therefore, we performed mediation analysis to verify whether the effect of registration date on the risk of the absence of information about the central contact person was significant per se or rather is a result of its relationship with the variable “Type of expanded access” (potential mediator). This analysis showed that the average causal mediation effect, that is the effect dependent on the mediator, was not statistically significant (*P*>.05; Table 6). By contrast, the average direct effect, that is the effect of the variable “Registration date” itself, was significant (*P*=.02; Table 6). Thus, the effect of the registration date on the risk of the absence of information about the central contact person is in itself statistically significant and is not a result of its relationship with the variable “Type of expanded access.”

We also found that the records of very few studies contained citations to publications (17/228, 7.5%) and links (31/228, 13.6%). Most of the links provided access to sponsors’ general web sites (12/31, 39%) or sponsors’ general policies of expanded access (8/31, 26%). As few as 3 links provided access to further information about a given expanded access program. The remaining links provided access to other related information.

Moreover, we examined the mode of registration of expanded access studies. Most of the studies (193/228, 84.7%) had the status “Available” in the first entry. This means that expanded access for a given intervention is available to patients. However, there were also several studies that, in the first entry, had the status “Temporarily not available” (16/228, 7.0%), “No longer available” (15/228, 6.6%), or “Approved for marketing” (1/228, 0.4%). According to the definitions of those statuses [11], each of them means that expanded access for a given intervention was available in the past. Thus, 32 studies (32/228, 14.0%) were *de facto* registered retrospectively. Remarkably, 21 studies (21/228, 9.2%) were not available to patients at any point. Three studies (3/228, 1.3%) had the status “Not yet recruiting” in the first entry; this is an unexpected finding because this status is typical of interventional clinical trials and not of expanded access studies [13].

**Table 6:** Mediation analysis to evaluate whether the effect of registration date on the risk of the absence of information about the central contact person is mediated by the type of expanded access.

Variable	Estimate	95% CI^a^	*P* value^b^
ACME^c^	0.05	–0.009-0.200	.18
ADE^d^	0.48	0.08-0.76	.02
TE^e^	0.53	0.19-0.77	<.001
PM^f^	0.05	–0.01-0.59	.18

^a^Quasi-Bayesian 95% CI.

^b^*P* value as determined by mediation analysis.

^c^ACME: average causal mediation effect.

^d^ADE: average direct effect.

^e^TE: total effect.

^f^PM: proportion mediated.

### Registration With ClinicalTrials.gov of the Published Expanded Access Studies

Another important problem that we addressed is the percentage of expanded access studies that has been registered with CT.gov. To that end, we examined papers reporting on results of expanded access treatment, with at least 1 center located in the United States, and published from 2014 through 2019. We excluded from this analysis studies that did not involve at least 1 US center because most expanded access studies registered with CT.gov were available to patients in the United States. Our inclusion criteria were met by 77 papers, and 70 different interventions were used in the published studies. In most of the studies, a drug was used (38/77, 49%), followed by a biologic (28/77, 36%) and medical device (11/77, 14%). The studies mostly concerned oncology (30/77, 39%), infectious diseases (10/77, 13%), cardiovascular diseases (10/77, 13%), and other medical specialties (27/77, 35%); 24 (24/77, 31%) studies were multinational, and 51 (51/77, 66%) were multicenter; 39 (39/77, 51%) studies were funded from industry sources, 9 (9/77, 12%) studies were funded from nonindustry sources, and the remaining 29 studies (29/77, 38%) did not receive any funding. The median number of patients was 23 (interquartile range, 3-149).

Only 21 papers (21/77, 27%) included an identifier typical of CT.gov. However, we assumed that some studies may have been registered without providing a relevant identifier in the corresponding publication. Therefore, using keywords from the publications, we examined whether CT.gov contains records matching the remaining 56 studies. We found that 12 of these (12/77, 16%) were actually registered. Thus, overall, 33 of the 77 published studies (43%) were registered with CT.gov.

To put these findings into broader context, we also evaluated the registration of clinical trials. In the first step, for each expanded access study, we tried to identify a corresponding clinical trial in PubMed of the same therapeutic intervention that involved at least 1 center located in the United States and was published in the same period of time. We identified 71 such trials (for the remaining 6 expanded access studies, there was no trial evaluating the same intervention). We found that 68 of 71 (96%) trials were registered with CT.gov. As few as 3 (3/71, 4%) trials were unregistered.

In addition, using logistic regression analysis, we identified the predictors of an expanded access study not being registered (Table 7). The following variables were entered into the analysis: funding source, number of patients, and whether the study was multicenter and multinational. Number of patients (sample) was a continuous variable. Funding sources (sponsors) were divided into 3 categories: (1) industry (when a study was at least in part funded by the pharmaceutical industry), (2) nonindustry (when a study was funded solely from nonindustry sources), and (3) none (when a study received no funding). A multicenter study was defined as a study performed at 2 or more centers. Studies performed in at least 2 countries were considered multinational. Logistic regression analysis was preceded by the evaluation of the multicollinearity of the variables. However, the GVIF value for each variable was between 1.1 and 1.4, indicative of a lack of multicollinearity (detailed data not shown).

In this analysis, we adjusted for multiple comparisons using Bonferroni correction. The adjusted level of statistical significance was set at *P*=.01. In the univariate analysis, studies with a lower number of participants and studies funded from nonindustry sources were more likely to be unregistered (*P*=.009 and *P*=.008, respectively; Table 7). We also found that single-center studies were less likely to be unregistered (*P*=.002; Table 7). However, none of the analyzed variables was a predictor of study nonregistration in the multivariate analysis (*P*>.01; Table 7).

While it was a lower number of patients that was a predictor of study nonregistration in the univariate analysis, a substantial percentage of studies involving a higher number of participants has not been registered either. In particular, 14 of 40 studies (35%) involving at least 20 participants have not been registered. Among the studies involving at least 100 participants, 5 of 22 (23%) have not been registered.

**Table 7:** Predictors of nonregistration of the published expanded access studies.

Variables		Univariate analysis		Multivariate analysis	
		Odds ratio (95% CI)^a^	*P* value	Adjused odds ratio (95% CI)^a^	*P* value
Sample		0.99 (0.99-0.99)	.009	0.99 (0.99-1.0)	.48
**Sponsor**					
	Industry	referent	N/A^b^	N/A	
	Nonindustry	1.6 (1.14-2.23)	.008	1.55 (1.08-2.24)	.02
	None	1.63 (1.06-2.51)	.02	1.6 (1.0-2.55)	.05
**Multicenter study**					
	Yes	referent	N/A	N/A	N/A
	No	0.7 (0.56-0.87)	.002	1.02 (0.73-1.43)	.87
**Multinational study**					
	Yes	referent	N/A	N/A	N/A
	No	0.9 (0.7-0.1.14)	.40	N/A	N/A

^a^Except for sample (which is a continuous variable), odds ratios and 95% CIs are shown related to the referent value for each of the remaining variables (sponsor, multicenter study, multinational study).

^b^N/A: not applicable.

## Discussion

Overall, our results show that the information about expanded access studies posted on CT.gov is quite fragmentary. Remarkably, less than one-half of expanded access studies performed in the United States have been registered with CT.gov. In some cases, this may result from noncompliance with the statutory requirement to register some expanded access studies with CT.gov. The FDAAA of 2007 obliges the responsible party of each applicable clinical trial being registered with CT.gov to specify whether the drug (or other intervention) evaluated in the trial is also available through expanded access [14]. If expanded access is available and the responsible party is the drug’s manufacturer, then the sponsor has to register the corresponding expanded access with CT.gov [8]. However, as shown by our results, some sponsors failed to meet this requirement. Other studies, especially those not linked with applicable clinical trials may remain unregistered because there are simply no regulations to mandate their posting on CT.gov.

A specific form of expanded access studies that are very likely to remain unregistered is small studies performed outside of expanded access programs. In general, expanded access has 2 different forms [10]. The first is expanded access programs (some of which are linked with applicable clinical trials). Those programs are launched by manufacturers of investigational treatments and open to patients who meet specific eligibility criteria. However, even in the absence of a specific program providing access to an investigational drug, a doctor can submit to the manufacturer a request for that drug for a limited number of patients under his or her care. Thus, some published expanded access studies (especially those involving a small number of participants) can be a result of not expanded access programs but treatment of single patients outside of a formal program. It is rather unlikely that studies performed outside of a formal expanded access program have been registered with CT.gov. Indeed, the univariate logistic regression analysis showed that studies with a lower number of participants are significantly less likely to be registered. While multivariate analysis did not confirm this finding, a subgroup analysis for the published studies involving a higher number of patients revealed that the proportion of unregistered studies was also considerable (eg, almost one-quarter for studies involving at least 100 participants). Thus, study nonregistration is an important problem even in the case of large expanded access programs.

For each of the 228 CT.gov-registered expanded access studies, we also evaluated the completeness of the posted information. We found that the records of many studies were incomplete, and the information that was missing most often included the detailed description of a study, facility information, central contact person, and eligibility criteria. The absence of some of those data depended on specific variables, especially the registration year. Specifically, study registration in 2017 significantly increased the odds of the absence of information about the central contact person. This may be associated with the FDAAA 801 Final Rule. It expanded requirements for the submission of clinical trial registration and results information to CT.gov [8]. While this rule was issued in September 2016, it became effective in January 2017, and the responsible parties were expected to be in compliance by April 18, 2017 [8]. We believe that at that time, many sponsors devoted most of their resources to ensure compliance with these regulations. This may have resulted in a situation where fewer resources could be devoted to expanded access (from a point of view of drug development, expanded access is certainly not as important as clinical trials). Therefore, for some responsible parties, it might take some time to improve standards of posting of information about expanded access studies on CT.gov.

However, we were unable to identify a single factor associated with an increased risk of nonposting of all types of data. In particular, in the multivariate analysis, whether the data were missing from records was not significantly associated with the funding source. Lack of some of those data, especially facility information, can result from the nature of expanded access. While some studies can be open to patients at specific medical centers, investigational drugs can also used by doctors residing in different locations [11]. In such cases, information about facility location would be irrelevant. However, we cannot see any justification for a lack of data such as central contact person or eligibility criteria (at least the main criteria should be listed). These are data that are particularly important for doctors and patients who seek access to unapproved tretments.

We also asked whether the absence of some of the required data is specific to expanded access studies or rather is a broader problem that pertains to clinical trials as well. We found that certain data were indeed missing from the records of some clinical trials. However, in the case of clinical trials, the scale of this problem was much smaller. Furthermore, we found that the percentage of unregistered clinical trials was very low compared with expanded access studies. Overall, CT.gov is a more complete source of information about clinical trials than expanded access studies, at least for studies performed in the United States.

An important question is whether, apart from CT.gov, there are other publicly available comprehensive sources of information about expanded access studies. Generally, information about investigational treatments can be obtained from a few different sources. The first of these are patient advocacy organizations’ web sites. However, it was shown that, while most of these present data on clinical trials, very few post any information about expanded access studies [15]. Another potential source of information is web-based expanded access navigators. In the United States, the primary navigator of that kind was developed by the Reagan-Udall Foundation for the FDA [16]. However, it posts data about single-patient expanded access only. Furthermore, data on specific expanded access studies posted by this resource are actually pulled from CT.gov [16]. Thus, if the information posted on CT.gov is incomplete, this navigator will not present complete data about single-patient expanded access studies. Furthermore, unlike CT.gov, the navigator posts no information about programs dedicated to intermediate-sized groups of patients and treatment IND/protocols. Overall, the navigator is a much less complete source of information about expanded access than CT.gov.

Some information about expanded access can be also found on pharmaceutical companies’ web sites [17]. In particular, in the United States, the 21st Century Cures Act of 2016 required manufacturers of investigational drugs to post key information about their general policies on evaluating and responding to expanded access requests [18]. However, this act does not explicitly require the manufacturers to post full listings of available expanded access studies. Rather, it obliges them to post a reference (eg, a hyperlink) to pertinent information on CT.gov.

Thus, currently CT.gov is the only primary source of information about specific expanded access studies, at least in the United States. In our opinion, CT.gov could be a very useful resource for patients and health care providers because it enables searching studies based on several criteria that are important in practice, including disease, intervention name, facility location, sponsor, or any combination thereof. However, some actions have to be undertaken to fully exploit the potential of CT.gov as a source of information about expanded access. First, CT.gov should implement some measures to promote submission of all data that are required when registering expanded access studies. Moreover, further research should be performed to evaluate what percentage of expanded access studies linked with applicable clinical trials have been registered with CT.gov. Such research could be performed on the data collected by the FDA. All expanded access studies in the United States have to be approved by the FDA [1]. Thus, by comparing the FDA’s data with CT.gov records, one could determine the extent of noncompliance with the statutory requirement to register expanded access studies linked with applicable clinical trials on CT.gov. If a substantial number of such studies have not been registered, then some measures should be introduced to enforce higher compliance. It is noteworthy that FDA-affiliated authors have already published some reports on expanded access based on the data contained in expanded access requests submitted to the FDA [19-21].

Moreover, we postulate registration with CT.gov of all expanded access studies available to patients in the United States (regardless of whether these are linked with applicable clinical trials). Since the registration of the latter category is not mandatory, a decision about possible registration rests with the manufacturer of the unapproved drug, biologic, or medical device. However, to ensure fair access of patients and health care providers to information about unapproved treatments, all planned expanded access studies should be registered. We hope that this article will raise awareness of this problem among sponsors of expanded access studies.

Our study also enabled us to make some interesting observations about the included expanded access studies. First, we found that most studies involved drugs used in oncology, neurology, and infectious diseases. In the case of oncology, this is likely caused by generally poor prognosis of patients with different kinds of cancer who have run out of approved treatments [22]. The high number of expanded access studies in neurology may be associated with the fact that some relatively frequent chronic neurological diseases (eg, Alzheimer’s disease) do not have effective treatments [23]. The high demand for unapproved treatments in infectious diseases can be a result of the development of resistance to approved drugs [24,25]. We also showed that only a small subset of studies in our cohort was funded by nonindustry sources. Therefore, in our view, in the future, noncommercial sponsors might consider more involvement in expanded access studies.

A limitation to our study is that we relied on data posted by CT.gov only and did not verify their validity. For instance, most of the included expanded access studies had the status “Available” in the first entry in the “History of Changes” field. However, theoretically, it is possible that in some of those studies, the actual start date was earlier and preceded the date of registration with CT.gov of a given study.

Another limitation is that we were unable to get access to some data about the factors that may impact the availability of information about expanded access. These include, in particular, the factors related to the sponsors of individual studies. For instance, the number of the staff members to prepare and enter relevant data into CT.gov records will likely affect the efficiency of the posting of information. Another important factor is whether the sponsor has any previous experience with posting on CT.gov of expanded access studies. Unfortunately, those data are generally unavailable, so we were unable to include them in our statistical analyses. We also could not analyze the exact number of patients that were treated in individual studies because CT.gov records do not contain relevant data. We hope that the publication of this article will encourage some sponsors to share their experiences with posting on CT.gov of expanded access studies.

In conclusion, CT.gov is the only primary, publicly available source of information about expanded access studies, especially those available in the United States. However, the data posted by this register are fragmentary, which is an important factor restricting access to information about investigational treatments. Introduction of the measures postulated in this paper will be beneficial to patients and health care providers who seek information about possibilities to use unapproved drugs, biologics, and medical devices.

## Abbreviations

aOR: adjusted odds ratio
CT.gov: ClinicalTrials.gov
FDA: Food and Drug Administration
FDAAA: FDA Amendments Act
GVIF: generalized variance inflation factor
IND: investigational new drug
OR: odds ratio

## References

[1] Expanded Access. Food and Drug Administration.

[2] Van Norman GA (2018). Expanded Patient Access to Investigational New Devices: Review of Emergency and Nonemergency Expanded Use, Custom, and 3D-Printed Devices. JACC Basic Transl Sci.

[3] Borysowski J, Górski A (2019). Compassionate use of unauthorized drugs: Legal regulations and ethical challenges. Eur J Intern Med.

[4] Nakada H, Takashima K (2019). Where Can Patients Obtain Information on the Preapproval Access Pathway to Investigational Treatment in Japan? A Survey of Patient Advocacy Organizations' Websites. Clin Pharmacol Drug Dev.

[5] da Silva RE, Lima E, Novaes M, Osorio-de-Castro C (2020). The High "Cost" of Experimental Drugs Obtained Through Health Litigation in Brazil. Front Pharmacol.

[6] Fountzilas E, Said R, Tsimberidou AM (2018). Expanded access to investigational drugs: balancing patient safety with potential therapeutic benefits. Expert Opin Investig Drugs.

[7] ClinicalTrials.gov.

[8] (2016). Clinical Trials Registration and Results Information Submission. Federal Register: the Daily Journal of the United States Government.

[9] Tse T, Fain KM, Zarin DA (2018). How to avoid common problems when using ClinicalTrials.gov in research: 10 issues to consider. BMJ.

[10] Caplan AL, Bateman-House A (2015). Should patients in need be given access to experimental drugs?. Expert Opin Pharmacother.

[11] Caplan AL, Teagarden JR, Kearns L, Bateman-House AS, Mitchell E, Arawi T, Upshur R, Singh I, Rozynska J, Cwik V, Gardner SL (2018). Fair, just and compassionate: A pilot for making allocation decisions for patients requesting experimental drugs outside of clinical trials. J Med Ethics.

[12] (2020). ClinicalTrials.gov Registration Data Element Definitions for Expanded Access. ClinicalTrials.gov PRS.

[13] (2020). ClinicalTrials.gov Protocol Registration Data Element Definitions for Interventional and Observational Studies. ClinicalTrials.gov PRS.

[14] Public Law 110–85. Food and Drug Administration Amendments Act of 2007.

[15] Folkers K, Leone S, Caplan A (2019). Patient advocacy organizations' information for patients on pre-approval access to investigational treatments. BMC Res Notes.

[16] Expanded Access Navigator. Reagan-Udall Foundation for the Food and Drug Administration.

[17] Jung E, Zettler PJ, Kesselheim AS (2018). Prevalence of Publicly Available Expanded Access Policies. Clin Pharmacol Ther.

[18] (2016). 21st Century Cures Act. United States Congress.

[19] Jarow JP, Lemery S, Bugin K, Khozin S, Moscicki R (2016). Expanded Access of Investigational Drugs: The Experience of the Center of Drug Evaluation and Research Over a 10-Year Period. Ther Innov Regul Sci.

[20] Jarow JP, Lemery S, Bugin K, Lowy N (2017). Ten-Year Experience for the Center for Drug Evaluation and Research, Part 2: FDA's Role in Ensuring Patient Safety. Ther Innov Regul Sci.

[21] Jarow JP, Moscicki R (2017). Impact of Expanded Access on FDA Regulatory Action and Product Labeling. Ther Innov Regul Sci.

[22] Wu T, Gu X, Cui H (2021). Emerging Roles of SKP2 in Cancer Drug Resistance. Cells.

[23] Li J, Huang Q, Chen J, Qi H, Liu J, Chen Z, Zhao D, Wang Z, Li X (2021). Neuroprotective Potentials of Panax Ginseng Against Alzheimer's Disease: A Review of Preclinical and Clinical Evidences. Front Pharmacol.

[24] Frieri M, Kumar K, Boutin A (2017). Antibiotic resistance. J Infect Public Health.

[25] Colpitts C, Baumert T (2016). Addressing the Challenges of Hepatitis C Virus Resistance and Treatment Failure. Viruses.

